# Two Cases of Ruptured Tubal Pregnancy

**Published:** 1903-03

**Authors:** Harold F. Mole

**Affiliations:** Assistant-Surgeon to the Bristol Royal Infirmary.


### TWO CASES OF RUPTURED TUBAL PREGNANCY.

BY

Harold F. Mole, F.R.C.S.,
*Assistant-Surgeon to the Bristol Royal Infirmary.*

Cases of extra-uterine gestation would appear to be of not infrequent occurrence, and many cases are from time to time reported, yet they are always of interest; and these two cases, admitted to the Royal Infirmary within a fortnight of one another, exhibit well the two varieties of rupture of the tube, and the satisfactory result of prompt surgical treatment in each.

It will be remembered that the Fallopian tube will not contain an impregnated ovum developing within its walls beyond the twelfth week. Some time previous to this, one of three things happens. It either aborts through the open ostium of the tube, or the tube ruptures into the peritoneal cavity, or gives way between the layers of the broad ligament. The two following cases are examples of the latter accidents.

**Case 1.**—Eliza S., æt. 34, admitted to the Royal Infirmary on the night of August 31st, 1902, complaining of severe pain in the abdomen, and passage of blood from the vagina. She had been married ten years, and had had two children, the younger of which was seven years old. She had not been pregnant for seven years. She had not menstruated for seven or eight weeks; but was always irregular, often going eight weeks without seeing anything. Three days previous to admission she had slight pain on the left side of the abdomen, which soon passed off; but the following day she was suddenly seized with severe pain in the lower abdomen, which doubled her up and made her vomit; and she had vomited several times during the last two days. On admission, the patient's appearance was expressive of great pain; her face was drawn and anxious. She was pallid and restless—pulse 120, temperature 99 F. The abdomen was extremely tender, and the parietes fixed and hard, especially over the left lower part. On vaginal examination, which was very painful, especially on the left of the uterus, the cervix was found soft, the os patulous, and the body of the uterus enlarged and tender,—all suggestive of pregnancy. Around the uterus was an ill-defined bogginess, but *no defined tumour*. There was secretion in the breasts.

On taking the results of the physical examination with the history, a pretty confident diagnosis of ruptured extra-uterine gestation, with bleeding into the pelvis and abdomen, was made, and operation performed shortly after admission. The usual incision between umbilicus and pubes was made. Blood could be seen through the peritoneum before that membrane was divided, and escaped in large quantity when the abdomen was opened. The hand was rapidly passed to the left side of the uterus, the symptoms having been most marked on that side, the tear in the tube was at once felt, and it and the ovary, which contained a recent corpus luteum, brought to the surface. These were ligatured off and removed. It was seen that the tube had given way close to its uterine end, and that the hole would admit the tip of the forefinger. The abdomen was then irrigated with hot saline solution, and all the blood and clot washed out. There was no sign of the embryo. The wound was then closed. The patient recovered without a bad symptom, and was discharged at the end of a month.

**Case 2.**—Ellen S., æt. 30, came some distance from the country as an out-patient to the Royal Infirmary, on September 13th, 1902, complaining of loss of blood from the vagina. She had been married twelve years, had one child ten years old, and had not been pregnant since. About five weeks previous to her coming to the Infirmary, she was suddenly attacked with pain in the right iliac fossa, and since that she has had two similar attacks—one four days ago, and one on the day of admission. She dated her last menstrual period to the time the first attack of pain came on, some five or six weeks previously. Since this time she had lost blood almost continuously, but more so when the pains came on. On examining the abdomen, a large tender swelling could be felt in the right iliac region, apparently arising from the pelvis. *Per vaginam* the os was small and soft, and pushed to the left; the body of the uterus enlarged and tender. To the right of the uterus a mass could be felt bimanually about the size of an orange, tender and firm, and apparently connected with the uterus itself. The breasts had been enlarging and becoming tender; but no secretion could be obtained. A diagnosis of ruptured tubal gestation was made, and the patient admitted and operated on on September 15th.

On opening the abdomen, a large tumour, as big as a fœtal head, was at once seen to fill the right side of the pelvis, lying immediately to the right of the uterus, which was large and flabby. Over the top of the tumour, the greatly distended Fallopian tube could be seen, extending from the uterine end in an outward and backward direction, where it was adherent to the bowel and mesentery. The rest of the tumour lay between the stretched layers of the broad ligament, little space intervening between it and the uterus. This narrow piece was ligatured off in sections from above downwards as far as possible, and the peritoneum then divided across the tumour. The front part of the tumour was then shelled out from the broad ligament. In separating the dilated Fallopian tube from the bowel, to which it was very closely adherent behind, this part of the sac gave way, some clear fluid escaping, followed by a fœtus. The cord was clamped and the fœtus removed. The enucleation was then proceeded with, below and in front, and this part of the sac, when nearly free, also ruptured, and a good deal of blood-clot, together with the placenta and its membranes escaped. Several vessels were ligatured in the ragged cavity left in the broad ligament, but, a considerable amount of oozing continuing, the cavity was stuffed with gauze, one en of which was brought out at the lower end of the abdomina wound, which was closed in the usual way.

The fœtus corresponded to the end of the fourth month. It was five inches in length, a male: generative organs well marked, head quarter whole length. Sac rather larger than a cricket ball. The placenta was fully formed.

The gauze was removed on the second day. A complete decidual cast of the uterus was passed on the fourth day. On the fifteenth day the breasts commenced secreting—rather an interesting point; and the patient was discharged cured on the thirtieth day.

The all-important point in these cases is the diagnosis; and to arrive at this correctly we must take into consideration most carefully every point in the history and symptoms. Now. tubal gestation is very liable to occur after long periods of sterility; and in both these cases the patients were young married women who had borne children, but who had remained sterile respectively for seven and ten years.

The next thing is, that on careful enquiry there is usually something abnormal about the menstruation—either one or more periods have been missed, or there has been some irregular hemorrhage, or hemorrhage may have been continuous from the last period up to the time of seeking advice. Again, shreds of decidua may have been observed with the discharge: and this is a point of great importance, for it must be remembered that in extra-uterine gestation, the uterus participates in the early changes of pregnancy: it enlarges, a decidua forms within it, and the cervix becomes soft and the os patulous. In the first case, the patient had seen nothing for two months, and then had an excessive discharge, accompanied by abdominal pain. In the second case, bleeding had been almost continuous since the last period, dated at about six weeks previously, when it came on accompanied by pain.

Thirdly, in a good many of the cases at any rate some of the signs of pregnancy are present. We have mentioned the missed periods; then there is the condition of the cervix and the uterus, the presence of milk in the breasts, and sometimes morning sickness. Some of these patients think themselves pregnant; more, I think, not. Neither of these patients believed herself to be pregnant. The uterus resembled an early pregnancy in each: milk was present in the breasts of one and not in those of the other. There was no definite morning sickness in either.

Now we have to differentiate between the intra-peritoneal and the extra-peritoneal rupture. In the former the symptoms are far more urgent—acute abdominal pain with collapse, and signs of faintness, not perhaps at first distinguishable from any other acute abdominal catastrophe, such as ruptured gastric ulcer, perforative appendicitis, twisted ovarian cyst, etc., etc.; but perhaps, from successive hemorrhages, the restlessness and pallor, with increased rapidity of pulse, will point more clearly to the fact that internal hemorrhage is going on. There is often very great pain in this condition; more so than one would expect perhaps, and most marked over the lower abdomen on one or other side. The vaginal examination is also very painful. It must be distinctly remembered that no tumour is present. Recently extravasated blood does not give rise to a tumour, and anything that one feels *per vaginam* may be very vague. Hence the great importance of careful consideration of history, as well as symptoms, in making the diagnosis.

In extra-peritoneal rupture, the symptoms are far less urgent, the tension of the layers of the broad ligament preventing any great extravasation of blood, and this bleeding not necessarily interfering with the life and subsequent growth of the fœtus. The three definite attacks of pain that occurred in the second case were no doubt caused by successive hemorrhages, yet the life of the fœtus was not interfered with. The rupture of the tube, in whatever position, is probably always accompanied by a discharge of blood from the uterus. In these cases a tumour is always present on one or other side of the uterus, and this is one of the distinguishing points between them and the intra-peritoneal cases. This tumour may be taken for an ovarian cyst, or a fibroid, or a tube distended from other causes; and here again, it is the association of important points in the history that is of help in the diagnosis. Whilst in some cases the diagnosis can be made with a fair degree of certainty, there are others in which all the evidence we can command leaves us in doubt.

We have finally to consider the question of operation in these cases. There appears to be a great probability that, if the ovum be apoplectic at the time of rupture, and if it be discharged from the tube, no further hemorrhage will take place, and if the initial hemorrhage be not sufficient to kill the patient, she may recover without operation; and this especially would apply to the cases of extra-peritoneal rupture. As, however, we have no means of knowing whether a blighted ovum has been discharged through the rupture, as delay might mean death from recurring hemorrhage, and as the results of these operations, even when the patients are *in extremis*, are so exceedingly satisfactory, the proper line of treatment is to operate at once when rupture into the peritoneum is diagnosed. Again, when we remember the enormous risk to the mother of a fœtus developing between the layers of the broad ligament, and the increasing dangers and difficulties of operation with each succeeding month of pregnancy, one will advise operation as early as the diagnosis can be made. It was lucky that the second case was caught just at the end of the fourth month. The operation was sufficiently difficult, but would have been much more so a month later. It will be noticed that the placenta was placed below the fœtus.

My remarks have been based almost solely on what could be learnt from the two cases reported, and I have purposely avoided making any reference to tubal abortion.

In conclusion, I must offer my thanks to Mr. Prichard and Mr. Munro Smith for kindly allowing me to publish these cases, which were respectively under their care at the time I was House Surgeon.

